# Comprehensive dataset of user-submitted articles with ideological and extreme bias from Reddit

**DOI:** 10.1016/j.dib.2024.110849

**Published:** 2024-08-22

**Authors:** Kamalakkannan Ravi, Adan Ernesto Vela

**Affiliations:** University of Central Florida, Orlando, USA

**Keywords:** Social networking (online), News media consumption, Reddit, Text classification, Context modeling, Topic modeling, Machine learning, Natural language processing

## Abstract

Our study aims to collect data to understand ideological and extreme bias in text articles shared across various online communities, particularly focusing on the language used in subreddits associated with extremism and targeted violence.

Initially, we gathered data from related online communities, specifically the r/Liberal and r/Conservative communities on Reddit, utilizing the Reddit Pushshift API to collect URLs shared within these subreddits. Our aim was to gather news, opinion, and feature articles, resulting in a corpus of 226,010 articles. We also curated a balanced subset of 45,108 articles and annotated 4000 articles to validate their relevance, facilitating understanding of language usage within ideological Reddit communities and insights into ideological bias in media content.

Expanding beyond binary ideologies, we introduced a new category termed “Restricted” to encompass articles shared in private or banned subreddits. This third category encompasses articles shared in restricted, privatized, quarantined, or banned subreddits characterized by radicalized and extremist ideologies. This expansion yielded a large dataset of 377,144 articles. Additionally, we included articles from subreddits with unspecified ideologies, creating a holdout set of 922,522 articles. In total, our combined dataset of 1.3 million articles collected from 55 different subreddits will assist in examining radicalized communities and providing discourse analysis in associated subreddits, enhancing understanding of the language used in articles shared within radicalized Reddit communities and offering insights into extreme bias in media content.

In summary, we collected 1.52 million articles to understand ideological and extreme bias, providing a comprehensive dataset that aids in understanding language usage within text articles posted in ideological and extreme Reddit communities.

Specifications Table

**Table:** 

Subject	Artificial Intelligence
Specific subject area	Computational Social Science, Social Computing, Computational Linguistics
Data format	Data 1: Raw, Sampled, Labeled Data 2: Raw and Labeled, Raw and Unlabeled
Type of data	Tables (.json)
Data collection	To compile text articles, we begin by extracting website links (URLs) from all posts within targeted subreddits. Utilizing the Pushshift Reddit API, we initiate data retrieval from the inception of each subredditʼs posts and extend the collection period until August 2021. Links originating from platforms such as YouTube and Imgur are filtered out, focusing solely on textual articles. Subsequently, employing the Beautiful Soup API, we scrape text content from the retained URLs. Throughout this process, measures are implemented to exclude empty or duplicate articles from our dataset. Furthermore, to ensure the inclusion of genuine text articles while eliminating irrelevant web content like video descriptions or copyright notices, a word-count threshold is applied.
Data source location	Reddit
Data accessibility	Part 1 has Data 1 (all) and Data 2 (Raw and Labeled Data - Restricted.json) Part 2 has Data 2 (Raw and Labeled Data - Liberal.json, and Conservative.json) and Data 2 (Raw and Unlabeled Data - first 40 of the 76 .json files) Part 3 has Data 2 (Raw and Unlabeled Data - remaining 36 of the 76 .json files) Data 1: Repository name: Reddit Ideological and Extreme Bias Dataset - Part 1 Data identification number: 10.17632/2tdr9sjd83.3 Direct URL to data: https://data.mendeley.com/datasets/2tdr9sjd83/3 Repository name: Reddit Ideological and Extreme Bias Dataset - Part 2 Data identification number: 10.17632/dxpp5983yb.1 Direct URL to data: https://data.mendeley.com/datasets/dxpp5983yb/1 Data 2: Repository name: Reddit Ideological and Extreme Bias Dataset - Part 2 Data identification number: 10.17632/dxpp5983yb.1 Direct URL to data: https://data.mendeley.com/datasets/dxpp5983yb/1 Repository name: Reddit Ideological and Extreme Bias Dataset - Part 3 Data identification number: 10.17632/f7knr8r94w.1 Direct URL to data: https://data.mendeley.com/datasets/f7knr8r94w/1 Direct URL to code to collect data: https://github.com/ADCLab/RedditIdeologyDB Instructions for accessing these data: Data and code are hosted publicly in Mendeley and GitHub, and can be downloaded by visiting the given links.
Related research article	Ravi, K., Vela, A. E., & Ewetz, R. (2022, December). Classifying the Ideological Orientation of User-Submitted Texts in Social Media. In 2022 21st IEEE International Conference on Machine Learning and Applications (ICMLA) (pp. 413–418). IEEE.

## 1 Value of the Data

• *Comprehensive Ideological Spectrum Analysis:* This dataset enables researchers to perform a nuanced analysis of ideological and extreme bias by providing a broad spectrum of articles from various Reddit subreddits. The inclusion of categories such as Liberal, Conservative, Restricted, and Undefined allows for a detailed examination of how different ideological perspectives and extremisms are expressed in online discourse, facilitating a deeper understanding of the ideological landscape on Reddit.
• *Benchmarking and Model Evaluation:* The large and diverse dataset serves as a valuable benchmark for evaluating and improving computational models used in natural language processing and machine learning. By offering labeled data from multiple ideological categories, it allows for the development and testing of models designed to detect and classify ideological bias, advancing research in automatic bias detection and content moderation.
• *Impact of Restricted Content:* The datasetʼs inclusion of articles from restricted or banned subreddits provides unique insights into the language and rhetoric used in more radicalized or extremist online communities. This aspect of the data is crucial for studying the propagation of extreme ideologies and their potential impacts, offering a window into how extremist content is communicated and received within isolated or covert online spaces.

## 2 Data Description

We conceptualize ideological affiliation as a summation of personally held community values [10,12]. With the goal of better understanding discourse and how different communities, each with their own common ideological affiliation, connect through news, our data collection centers on the social media website Reddit. Our approach to understanding ideological bias in the news sets us apart from common data sources such as surveys or crowd-sourced datasets, which typically investigate political ideology and media bias. Some prior work uses platforms such as Amazon Mechanical Turk or conventional surveys like Pew Research [1]. Other studies rely on datasets like media bias datasets [2], the Congressional Tweets Dataset [3], Twitter fake-news influence datasets [4], biased Wikipedia articles [5], and Facebook News outlets [6]. Some surveys do not assess the articles themselves; instead, they gauge the ideological bias of the source. These datasets often include content from diverse users with varying ideologies. Our approach is unique; we do not separate data collection and labeling.

For the first dataset (Data 1), specifically, we are interested in the *r/Liberal* and *r/Conservative* communities where members actively share and discuss news articles and blog posts. Towards this goal we have collected news articles posted to each subreddit to better understand ideological expression through the shared news articles, and more specifically, if and how ideological bias may be expressed in the news articles. This method effectively captures the values and perspectives of these specific online communities. A summary of the collected data is provided in Table 1.

**Table 1:** Open-sourced data details for Data 1.

Folder	Filename	Description
Raw - all articles	1. Liberal.json 2. Conservative.json	22,554 liberal and 203,456 conservative articles
Sampled class-balanced articles	1. Liberal.json 2. Conservative.json	22,554 liberal and 22,554 conservative articles
Annotated articles	1. Liberal.json 2. Conservative.json	2000 liberal and 2000 conservative articles

In addition, this dataset was limited in its ability to capture ideological extremism due to its binary classes, which, in turn, hindered its ability to demonstrate the effectiveness of its approach. To overcome this limitation, we have expanded (Data 2) the dataset in two keyways. Firstly, we have included text articles from additional Liberal and Conservative ideology identifying subreddits. Secondly, we have introduced a new category referred to as the “Restricted class.” This category enables us to explore radicalized and extremist ideologies by incorporating articles from restricted, privatized, quarantined, or banned subreddits. Table 2 provides a summary of this comprehensive data.

**Table 2:** Open-sourced data details for Data 2.

Folder	Filename	Description
Raw and Labeled Data	1. Liberal.json 2. Conservative.json 3. Restricted.json	72,488 articles (from 6 subreddits) in the liberal class, 79,573 articles (from 6 subreddits) in the conservative class, and 225,083 articles (from 16 subreddits) in the restricted class.
Raw and Unlabeled Data	1. 76 .json files with subreddits’ name	922,522 articles from 25 subreddits.

## 3 Experimental Design, Materials and Methods

To collect the articles contained within the corpus, we gather identification numbers (*IDs*) and web addresses (*URLs*) of submissions from the subreddits, starting from the first post date of each subreddit up to August 10, 2021 using the Pushshift Reddit API [7]. Many of the *URLs* lead to non - text websites like YouTube and Imgur, which are excluded and outside the scope of the resulting corpus. We utilize the Beautiful Soup API [8] to extract text content from the remaining *URLs* and then perform data processing to eliminate empty responses, and duplicates articles from different *URLs* (duplicate webpage responses are common when sharing the original and short-form *URLs* using *URL* shortening tools like Bitly).

Further, we refine the corpus by eliminating non-relevant webpage texts which are unlikely to be news articles or posts such as “404 error” messages or copyright statements. To achieve this, we apply a simple word-count threshold to identify and exclude documents that are unlikely to be articles. This process ensures that our dataset contains relevant and meaningful articles, enhancing its quality and usefulness.

To establish an appropriate word-count threshold, first we divided articles into 20 bins based on their word-count distribution. Next, 100 articles from each category are selected at random for human annotation to label the documents as a verified article or not. The annotation process was conducted by a graduate student using the open-source annotation tool Doccano [9]. Subsequently, established word count threshold to exclude non articles.

For collecting the first dataset (Data 1), we followed the above-described method and collected 22,554 liberal articles and 203,456 conservative articles. The resulting corpus presented a class imbalance between r/Liberal and r/Conservative spanning the years 2008–2021. For classifier development using a balanced dataset, we retained 22,554 *r/Liberal* articles and sampled an equal number from *r/Conservative*, maintaining the same daily rates. In this data collection, we labeled 4000 articles into genuine article or not for establishing word count threshold.

Next to build the expanded dataset (Data 2), we collect articles from the Liberal, Conservative, and Restricted category subreddits that align with similar beliefs and interests. Further, we also include a separate holdout dataset collected from various subreddits, representing a mix of overt, vague, and undefined ideologies, as showed in Table 3.

**Table 3:** Open-sourced data details.

Ideology	Subreddit	No of articles by subreddit	No of articles by ideology
Liberal	1. progressive 2. socialism 3. obama 4. occupywallstreet 5. neoliberal 6. democrats	1309 1607 7985 11,326 18,162 32,099	72,488
Conservative	1. askaconservative 2. NolibsWatch 3. Romney 4. neoconNWO 5. Republican 6. Conservative	14 112 643 1705 30,005 47,094	79,573
Restricted	1. DylannRoofInnocent 2. alllivesmatter 3. pol 4. Physical_Removal 5. nazi 6. WhiteRights 7. ZOG 8. NationalSocialism 9. paleoconservative 10. 911truth 11. tea_party 12. HBD 13. CringeAnarchy 14. uncensorednews 15. new_right 16. The_Donald	3 4 253 298 345 759 821 1239 1375 1805 1851 2580 5056 21,876 38,353 148,465	225,083
Undefined (Holdout)	1. bluelivesmatters 2. NeutralPolitics 3. ShitPoliticsSays 4. EnoughObamaSpam 5. LibertarianSocialism 6. LibertarianPartyUSA 7. LibertarianLeft 8. BlackLivesMatter 9. EnoughPaulSpam 10. prolife 11. overpopulation 12. antiwar 13. EnoughLibertarianSpam 14. prochoice 15. alltheleft 16. DescentIntoTyranny 17. moderatepolitics 18. lostgeneration 19. Anarcho_Capitalism 20. Liberal 21. EndlessWar 22. climateskeptics 23. ChapoTrapHouse 24. politics 25. news	6 142 148 532 604 974 1070 1195 2066 2871 3064 3162 3212 3989 6014 6074 6939 10,309 14,804 16,399 17,842 17,950 26,607 386,797 389,752	922,522

To ensure the inclusion of only authentic text articles, while excluding unrelated webpage content like video descriptions and copyright templates, we introduce a word-count threshold. The threshold was determined by conducting annotations on a subset of 600 articles from the *CringeAnarchy* subreddit. All *CringeAnarchy* articles were divided into 12 bins based on word count, and 50 articles were randomly selected from each bin. The annotations were carried out using Doccano [9], which is an open-source web-based annotation tool. Our objective is to identify a word count threshold at which 90 % of the articles can be classified as “long text.” Building on first dataset collection, we set that a word limit of 300 is suitable for categorizing articles as long text. However, certain subreddits failed to meet these criteria, leading to the exclusion of the *Socialism_101* and *far_right* subreddits from our data collection.

Subsequently, the remaining corpus is categorized, as presented in Table 3, based on whether the respective subreddits express a clear ideology on the Reddit platform. Articles originating from subreddits with explicitly stated ideologies are categorized into three groups: 72,488 articles in the Liberal class, 79,573 articles in the Conservative class, and 225,083 articles in the Restricted class. Conversely, articles from subreddits lacking a clearly defined ideology, including those with implicit or explicit ideologies, are merged to form a holdout dataset comprising 922,522 articles. This holdout dataset will serve as a case study.

## Ethics Statement

We recognize that classification of text articles [10,13] using self-identifying subreddit data is not representative of the broader conservative and liberal communities within the United States, especially since Reddit users skew with regards to many key demographic features (esp. age, gender, education, and political ideology [11]). This distinction is also important when one considers that not all Reddit users are from the United States. Additionally, up until August 10, 2021, the Pushshift API allowed access to Reddit data, including comments and posts. Further, we obtained ethical clearance from our Institutional Review Board (IRB) at the University of Central Florida, receiving a “Not Human Subjects Determination” for our project. This determination signifies that our research does not involve or interact with human subjects in any way, and as such, it falls outside the purview of human subjects research regulations. This ethical clearance reinforces our commitment to responsible and ethical research practices.

## CRediT authorship contribution statement

**Kamalakkannan Ravi:** Data curation, Methodology, Software, Visualization, Writing – original draft. **Adan Ernesto Vela:** Conceptualization, Methodology, Writing – review & editing, Resources, Funding acquisition.

## Data Availability

Reddit Ideological and Extreme Bias Dataset - Part 1 (Original data) (Mendeley Data). Reddit Ideological and Extreme Bias Dataset - Part 2 (Original data) (Mendeley Data). Reddit Ideological and Extreme Bias Dataset - Part 3 (Original data) (Mendeley Data).
